# Oral Collagen Peptides and Vulvovaginal Radiofrequency Therapy for Genitourinary Syndrome of Menopause: A Pilot Randomized Study

**DOI:** 10.3390/jcm14113656

**Published:** 2025-05-23

**Authors:** Alessandro Tafuri, Andrea Panunzio, Michela Tricarico, Ezio Michele Tricarico, Claudia Rita Mazzarella

**Affiliations:** 1 Department of Urology, “Vito Fazzi” Hospital, Piazza F. Muratore 1, 73100 Lecce, Italy; panunzioandrea@virgilio.it; 2 Gynecology, “Amalthea” Diagnostic Center, Viale Marche 27, 73100 Lecce, Italy; michela.tricarico.00@gmail.com (M.T.); ezio.tricarico@libero.it (E.M.T.); 3 Department of Obstetrics and Gynecology, “Giuseppe Tatarella” Hospital, Via Trinitapoli, 71042 Cerignola, Italy; claudiamazzarella77@gmail.com

**Keywords:** bioactive collagen peptides, BCP^®^, genitourinary syndrome of menopause, GSM, ultra-low-molecular-weight hyaluronic acid, ULMW-HA

## Abstract

**Background/Objectives**: Genitourinary syndrome of menopause (GSM) encompasses a variety of symptoms associated with estrogen deficiency, affecting the genitourinary tract. Effective management often requires a multifaceted approach. Although radiofrequency (RF) treatment has been explored as a non-hormonal intervention for GSM, evidence remains limited and inconclusive. Oral collagen peptides have demonstrated systemic tissue benefits in dermatological studies, but with effects that are not yet well understood in the context of GSM. This pilot study investigated whether combining RF with an oral supplementation containing specific bioactive collagen peptides and ultra-low-molecular-weight hyaluronic acid would provide superior symptom relief compared to RF alone in women with GSM. **Methods**: Twenty menopausal women were randomized into two groups: Group 1 (n = 10) received vulvovaginal RF treatment every two weeks for two months; Group 2 (n = 10) received the same RF treatment alongside daily oral supplementation for four months. Subjective symptoms, objective signs, and vaginal pH were assessed at baseline (T0), post-RF treatment (T1), and three months post-RF treatment (T2), employing a two-way repeated-measures ANOVA to assess differences between groups over time. **Results**: Both groups showed substantial improvements in all the clinical parameters evaluated at T1 and T2 compared to T0. However, the magnitude of such improvements was higher among patients from Group 2, who achieved better outcomes at T2 compared to patients from Group 1, with differences being statistically significant regarding subjective symptoms (*p* < 0.001), objective signs (*p* < 0.001), and vaginal pH (*p* = 0.015), thus demonstrating the sustained benefits of the combination therapy over RF treatment alone during the follow-up time. **Conclusions**: Combined treatment with vulvovaginal RF and food supplements improved the signs and symptoms of GSM, and compared to RF treatment alone, it enhanced and maintained the benefits in a three-month follow-up period.

## 1. Introduction

Genitourinary syndrome of menopause (GSM) is a chronic progressive condition resulting from estrogen deficiency that affects the vulvovaginal and lower urinary tract tissues [1,2]. Previously known as vulvovaginal atrophy, GSM may also occur following oncological treatments such as chemotherapy, pelvic radiotherapy, or anti-estrogen therapies including tamoxifen and aromatase inhibitors [3]. GSM symptoms commonly include vaginal dryness, burning, itching, irritation, dyspareunia, and abnormal discharge, and are frequently associated with urinary complaints such as dysuria, urgency, incontinence, and recurrent urinary tract infections [4,5]. These symptoms can have a significant negative impact on the quality of life, emotional well-being, and sexual function of postmenopausal women [4,5].

Although GSM affects up to 50% of postmenopausal women, it is frequently underdiagnosed and undertreated due to communication barriers and the normalization of symptoms as a part of aging [1]. Treatment options include local and systemic hormonal therapies, non-hormonal topical agents, and physical interventions. Hormonal treatments, such as local estrogens and selective estrogen receptor modulators are typically considered as first-line treatments. Non-hormonal alternatives, such as moisturizers, hyaluronic acid (HA) formulations, and pelvic floor muscle training, may be appropriate for women with contraindications to hormones. However, their efficacy varies, and supporting evidence is limited [1,5].

Energy-based therapies such as lasers and radiofrequency (RF) have gained interest as potential non-pharmacological options. RF is hypothesized to induce neocollagenesis and improve tissue quality through controlled thermal stimulation. However, clinical data supporting RF use in GSM remains limited, and the transient nature of these benefits underlines the need for evaluating long-term outcomes. As such, further research is needed to assess the effectiveness and safety of RF in this setting [5,6].

In parallel, oral supplementation with collagen peptides and HA has been explored in other medical fields, particularly dermatology. Specific bioactive collagen peptides (BCPs^®^) have demonstrated the ability to stimulate fibroblast activity and promote extracellular matrix synthesis, contributing to improved skin elasticity and hydration in randomized controlled trials [7,8,9,10,11]. Ultra-low-molecular-weight HA (ULMW-HA) may be characterized by enhanced absorption, thus providing systemic hydration benefits [12]. Despite this biological rationale, no studies to date have investigated the use of these components in the treatment of GSM. To address this gap, we conducted a randomized, controlled pilot study to evaluate the clinical effect of a food supplement containing specific BCPs^®^ (2.5 g), ULMW-HA (75 mg), a botanical powder mix (*Astragalus membranaceus* and *Centella asiatica*; 250 mg) and a nucleotide blend (5′-AMP, 5′-CMP, 5′-UMP, 5′-GMP, 5′-IMP; 100 mg) combined with vulvovaginal RF treatment. We hypothesized that the combined approach would more effectively enhance vulvovaginal eutrophism and alleviate GSM symptoms, compared to vulvovaginal RF treatment alone. Additionally, we anticipated that this combination would be better tolerated and produce a longer-lasting effect.

## 2. Materials and Methods

### 2.1. Study Population

This prospective randomized study was conducted between January and June 2023 and included 20 volunteer post-menopausal women diagnosed with GSM. All participants provided written informed consent for enrollment, data collection, and analysis. Inclusion criteria included a recent negative cervical–vaginal cytology (within 12 months) and a negative urine culture. Exclusion criteria consisted of active vulvovaginal or urinary infections, abnormal uterine bleeding, and recent pelvic surgery. Patients were randomly assigned (1:1) to two groups using a computer-generated randomization list: Group 1 received vulvovaginal RF treatment only, while Group 2 received RF combined with daily oral supplementation.

### 2.2. Radiofrequency Treatment

All participants received four vulvovaginal RF sessions spaced two weeks apart. Treatments were performed using bipolar, temperature-controlled RF (SECTUM, Nrose, Neauvia, Manno, Switzerland) operating at 480 kHz. The standard setting used for all patients involved continuous energy delivery at 25–30 W. These were the only settings used in the study.

**Figure 1:**
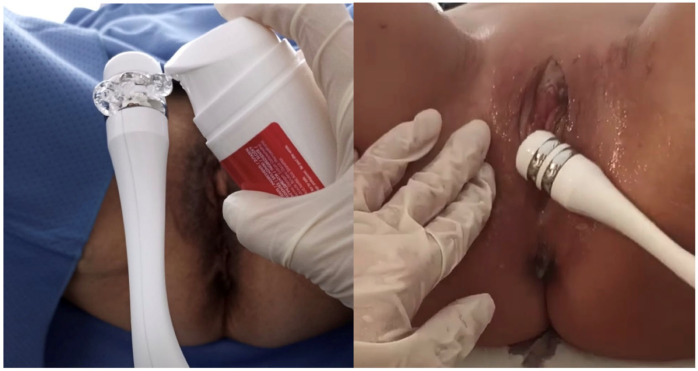
Vulvovaginal treatment session using bipolar radiofrequency at 480 kHz. A generous amount of HA in the form of gel was applied to the disposable handpiece, serving both as a moisturizer and a conductor.

Before each session, an HA-based conductive gel was applied to a disposable handpiece. The probe was inserted into the vaginal canal, and RF was applied in pulse mode (500 ms) using slow longitudinal movements for approximately 15–20 min, after reaching an internal temperature of 38 °C (not exceeding 40 °C). Following the vaginal phase, the same probe was used externally on the vulvar region for an additional 10 min, also in pulsed mode and with gel application. All treatments were performed by a trained gynecologist following a standardized protocol (Figure 1).

### 2.3. Oral Supplementation Treatment

Only participants in Group 2 received a commercially available food supplement containing the following daily dose: 2.5 g of BCP^®^ (Gelita AG, Eberbach, Germany), 75 mg of ULMW-HA (3–10 kDa, TS-Biotech Co., Ltd., Weifang, China), 250 mg of a botanical powder mix (*Astragalus membranaceus* and *Centella asiatica*, NuLiv Science, Inc., Brea, CA, USA), and 100 mg of a nucleotide blend (5′-AMP, 5′-CMP, 5′-UMP, 5′-GMP, 5′-IMP, Prosol S.p.A., Madone, Italy). The supplement was administered once a day for four months.

### 2.4. Timepoints and Evaluation of Parameters

Data collected at baseline included: age (years), body mass index (BMI; kg/m^2^), menopause age, parity, marital status, educational level, and comorbidities.

The study included three assessment points. T0, baseline, marked the beginning of the study and the start of the RF treatment for both groups, as well as the start of the oral supplementation for Group 2 only. T1 occurred two months later, at the completion of the RF treatment protocol (four sessions administered every two weeks). T2 was scheduled three months after T1, corresponding to five months from baseline; during this period, only Group 2 continued to receive oral supplementation, thus completing a total of four months of daily intake. No treatment was administered to Group 1 after T1. This design allowed us to assess the immediate effects of the RF treatment and to explore the potential sustained benefits of the combination therapy at follow-up.

**Figure 2:**
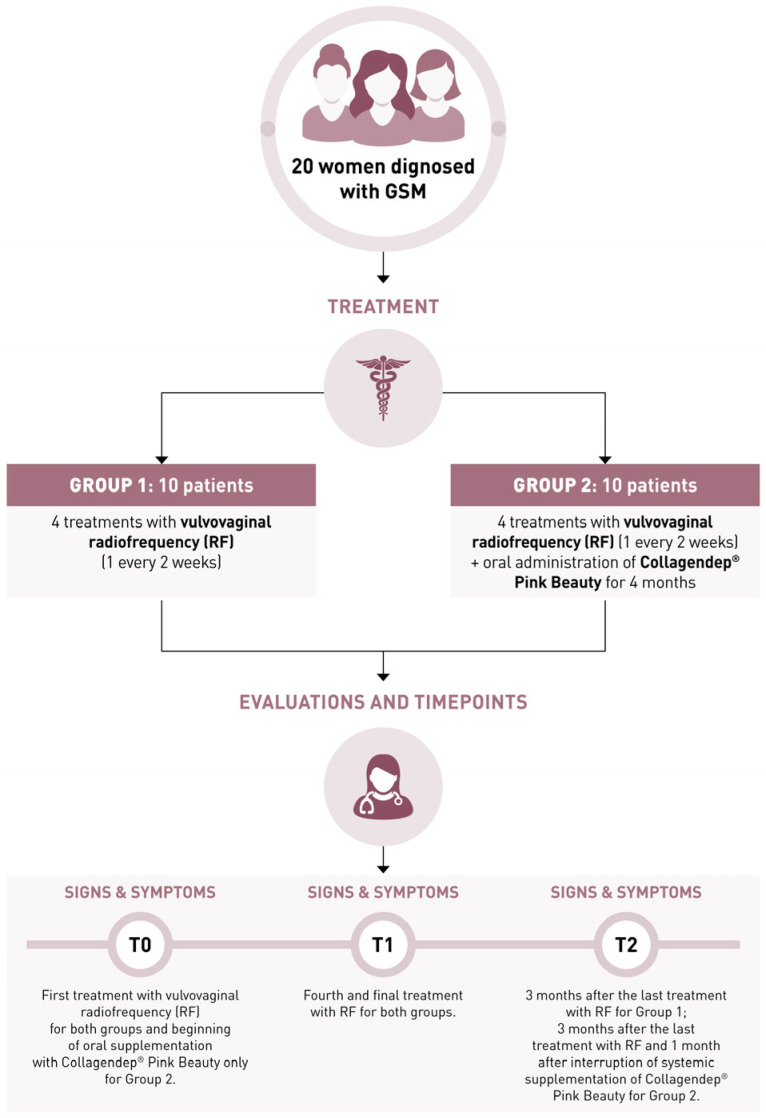
Flow chart illustrating study design, time points, and outcomes measured.

Clinical outcomes were assessed using a structured checklist covering subjective symptoms (vaginal dryness, dyspareunia, irritation/burning/itching, dysuria, and post-coital bleeding), objective signs (tissue elasticity, vaginal folds, fluid secretion, epithelial thickness, moisture, and color of the vaginal tissue), and vaginal pH. Each sign and symptom was rated using a four-tier scale (0 = absent/normal, 1 = mild, 2 = moderate, 3 = severe) in line with previous approaches used in studies investigating RF or laser therapy in GSM [13,14,15]. Objective signs were evaluated during gynecological examination through visual inspection and gentle palpation. Although the Vaginal Health Index is more commonly used in the literature, we chose this more granular checklist to allow for item-specific tracking, which we considered more appropriate given the exploratory and small-scale nature of this pilot study. The total symptoms score (0–15) and total signs score (0–18) were obtained by summing the individual item scores. Vaginal pH was measured with standardized pH Test Strips (Just Fitter, Vaginal Health Series), and scored as follows: >6.1–1, from 5.6 to 6.0–2, from 5.1 to 5.5–3, from 4.6 to 5–4, and <4.5–5. The primary endpoint was the change in total symptoms and sign scores over time. T2 was used to evaluate the persistence of treatment effects and the potential sustained benefit of oral supplementation. Adverse events, product tolerance, and compliance were also monitored throughout the study. The flow chart illustrating the study design, outcomes, and time points is shown in Figure 2.

### 2.5. Statistical Methods

Descriptive statistics included frequencies and proportions for categorical variables; medians and interquartile ranges (IQRs) were reported for continuously coded variables. To assess changes over time and between groups, a two-way repeated-measures ANOVA was conducted for each outcome variable (total median subjective symptoms score, total median objective signs score, and median vaginal pH) with time (T0, T1, T2) as the within-subjects factor and group (RF vs. RF + food supplement) as the between-subjects factor. The main effects of time and group, as well as the time × group interaction, were evaluated. Sphericity was tested using Mauchly’s test, and, where necessary, Greenhouse–Geisser corrections were applied. Following significant interactions, post hoc independent-sample *t*-tests were performed to compare the two groups at each time point. All tests were two-sided with a significance level set at *p* < 0.05. The R software environment for statistical computing and graphics (version 4.1.2, R Foundation for Statistical Computing, Vienna, Austria) was used for all analyses (ez, tidyverse, and broom packages) [16].

## 3. Results

**Figure 3:**
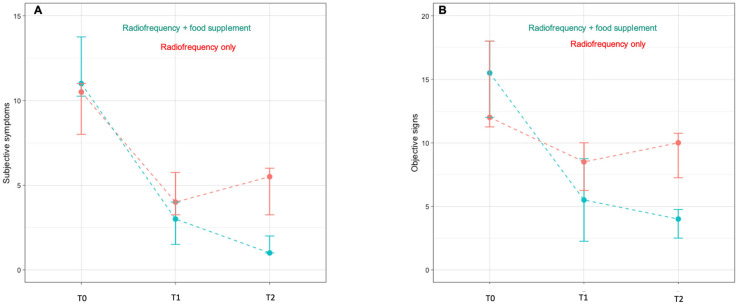
Line plots depicting median (IQR) total scores for subjective symptoms (**A**) and objective signs (**B**) of patients from Group 1 (RF only) and Group 2 (RF and food supplementation) at T0, T1, and T2.

Baseline demographics and clinical characteristics of the participants, stratified by treatment group (Group 1: RF alone; Group 2: RF and oral supplementation) are presented in Table 1. Clinical outcome scores across the three time points (T0, T1, and T2) are shown in Table S1 and summarized in Figure 3.

Data from three outcome variables (total subjective symptoms score, total objective signs score, and vaginal pH) were analyzed for the two groups of participants across three time points. The repeated-measures ANOVA revealed a highly significant main effect of time for all variables (*p* < 0.001), indicating an overall improvement over the course of treatment. Specifically, for both total subjective symptoms and objective signs scores, a significant group × time interaction was observed (*p* < 0.001), suggesting that the pattern of change differed between groups. In contrast, no significant interaction was found for pH (*p* = 0.117), although a significant time effect persisted (*p* < 0.001).

Post hoc comparisons showed that at T0 and T1, the differences between groups were not statistically significant. Specifically, at baseline (T0), both groups had comparable scores. The median total symptoms score was 10.50 (IQR: 8.00, 11.00) in Group 1 and 11.0 (IQR: 10.25, 13.75) in Group 2 (*p* = 0.2). The median total signs score was 12.00 (IQR: 11.25, 18.00) in Group 1 and 15.50 (IQR: 12.00, 18.00) in Group 2 (*p* = 0.4). The median vaginal pH score was 1.5 (IQR: 1.0, 2.0) in both groups (*p* = 0.9). Following the RF treatment (T1), both groups showed clinical improvement. In Group 1, the median total symptoms score decreased to 4.00 (IQR: 3.25, 5.75), the median total signs score decreased to 8.5 (IQR: 6.25, 10.0), and the median vaginal pH score increased to 4.5 (IQR: 4.0–5.0). Group 2 showed a further reduction in the median total symptoms score to 3.00 (IQR: 1.50, 4.00) and median total signs score to 5.50 (IQR: 2.25, 8.75), while the median vaginal pH score increased to 5.0 (IQR: 4.0–5.0). However, none of these differences between groups reached statistical significance at T1, suggesting that RF alone was associated with short-term clinical improvements. At follow-up (T2), three months after the last RF session, Group 1 exhibited a partial loss of benefit, with the median total symptoms score rising to 5.50 (IQR: 3.25, 6.00) and the median total signs score rising to 10.00 (IQR: 7.25, 10.75). In contrast, Group 2, who continued oral supplementation, showed further improvement, with the median total symptoms score dropping to 1.00 (IQR: 1.00, 2.00) and the median total signs score dropping to 4.00 (IQR: 2.50, 4.75)—both statistically significant compared to Group 1 (*p* < 0.001). The median vaginal pH scores also remained higher in Group 2 (4.5; IQR: 4.0, 5.0) vs. Group 1 (4.0; IQR: 3.0, −4.0) (*p* = 0.015).

Overall, these results suggest that RF treatment alone led to a measurable but transient clinical benefit, which tended to decline after treatment discontinuation. The addition of oral supplementation appeared to not only enhance, but also sustain the therapeutic effect over time. Figure 3 supports these findings, showing the respective trajectories of the symptoms and signs scores; however, it should be interpreted as illustrative, and its claims should be viewed in light of the small sample size and lack of a placebo group.

No adverse effects related to the oral supplementation were reported.

## 4. Discussion

In this pilot study, four sessions of vulvovaginal RF therapy led to short-term improvements in GSM symptoms, clinical signs, and vaginal pH. However, the persistence and magnitude of these effects differed between the two study groups. While RF alone (Group 1) provided an initial improvement, these benefits diminished over time. In contrast, the group receiving both RF and oral supplementation (Group 2) showed continued improvement at the three-month follow-up. These differences reached a statistical significance at T2, supporting a potential role for the combined approach. Importantly, although both groups improved after RF therapy, the sustainability of this improvement was only seeded in the group receiving oral supplementation. This suggests that RF alone, while effective in the short term, may not ensure lasting benefits for GSM. This interpretation aligns with existing regulatory and scientific concerns regarding the durability of energy-based interventions [17]. Nonetheless, the improvements observed in Group 1 support the conclusion that RF does exert a short-term effect. Figure 3 illustrates the trends in symptoms and signs scores across time points. While the trends are consistent with the statistical results, the figure should be considered exploratory due to the limited sample size. Its interpretation must be cautious, and its primary role is to visualize the differences rather than to independently substantiate efficacy claims.

From a mechanistic perspective, temperature-controlled RF delivers heat to stimulate collagen remodeling and tissue perfusion [18,19,20]. When used alone, these effects may be transient. Within non-hormonal therapies, nonprescription vaginal topical tightening products, such as gels and creams, have been suggested as alternative/concomitant options for GSM treatment. However, these therapies are often not well received by patients and cause severe disruption to the vaginal ecosystem, which can lead to mucosal erosion, increased vaginal discharge, and a higher rate of infections [21]; patient dissatisfaction largely stems from these adverse outcomes, which highlights a potential rationale for exploring alternative strategies to improve tolerability and adherence in GSM management. Treatments aimed at restoring the metabolism and function of connective tissue, the most important of which is bio-stimulation—activating fibroblast anabolic functions and particularly enhancing type III collagen, elastin, and HA production from their precursors—are considered an innovative approach according to the principles of antiaging medicine [22]. However, few studies support the application of these treatments as therapeutic strategies, and to the best of our knowledge none of them support the oral use of supplements for vulvovaginal health during GSM [23]. There is a need to explore therapeutic alternatives aimed at amplifying and extending the efficacy of intravaginal energy-based methods [24].

**Figure 4:**
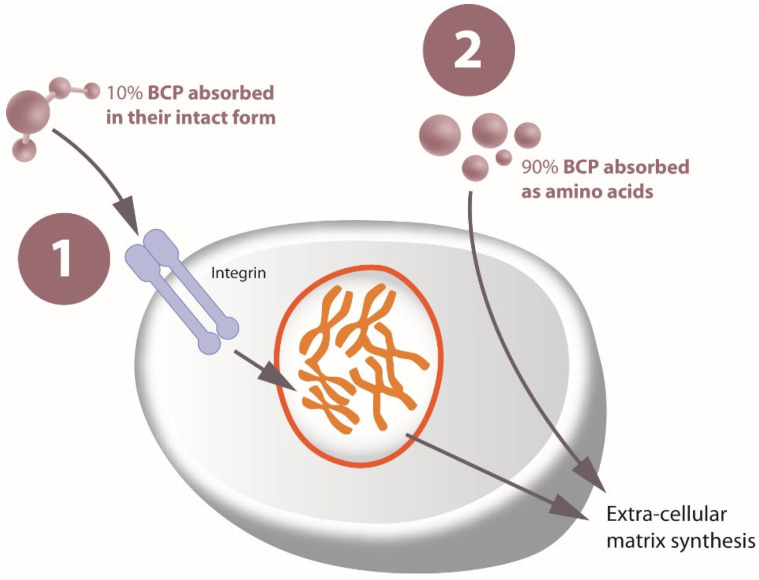
The BCPs^®^ contained in the food supplement stimulate connective tissue cells through receptor interaction. The amino acids resulting from their digestion are also important, as they provide the substrate for the synthesis of new connective tissue proteins once the target cells have been directly stimulated by the BCPs^®^. This is possible thanks to the unique molecular profile of the BCPs^®^, which have an average molecular weight of 2 kDa, and greater efficacy in stimulating the synthesis of the extracellular matrix compared to other collagen peptides with similar specifications (adapted from Siebert et al., 2010 [26]; Oesser and Seifert, 2003 [27]).

In this study, the addition of oral supplementation containing BCPs^®^, ULMW-HA, and other functional components appears to amplify and prolong the tissue response initiated by RF. These BCPs^®^, derived from bovine skin, have a unique stimulatory effect that is independent of native collagen types I, II, and III; this effect is achieved through a complex multistep process that requires a specific enzymatic hydrolytic step and denaturation of the original collagen. This process results in a distinctive mass peak fingerprint, as identified by matrix-assisted laser desorption ionization mass spectrometry. The final BCP^®^ composition is characterized by a specific amino acid profile, where glycine, proline, and hydroxyproline together represent 46.8% of the total amino acid weight, and where the average molecular weight is 2.0 kD. These BCPs^®^, when administered orally, are absorbed both as intact peptides and as free amino acids. While free amino acids provide building blocks for the formation of dermal extracellular matrix proteins and for epidermal structure, the collagen peptides act as bioactive messengers, activating different signaling pathways and stimulating human fibroblasts to synthetize collagen, proteoglycans, and elastin [8]. Although not a direct demonstration, Figure 4 provides a conceptual overview of the dual absorption pathways hypothesized for BCPs^®^. Approximately 10% of peptides are absorbed intact and interact with cell receptors, while 90% are digested into amino acids, contributing to extracellular matrix formation. The biological plausibility of this mechanism is supported by prior clinical studies showing that BCPs^®^ improve skin elasticity and extracellular matrix composition [7,8,9,10,11]. In two prospective, randomized, placebo-controlled clinical trials, Proksch et al. demonstrated that women who consumed 2.5 g/day of these specific BCPs^®^ for 8 weeks experienced improvements in skin elasticity, a reduction in wrinkle volume, and an increase in the content of collagen I and elastin in their skin [7,8]. Other clinical studies using the same specific BCPs^®^ showed their efficacy in wound healing [9], nail growth and strength [10], hair thickening [11], and cellulite morphology [25].

On the other hand, hyaluronan is a glycosaminoglycan polymer typically found as a high-molecular-mass polymer (several thousand kDa). It is a crucial component of the extracellular matrix and plays a variety of physiological roles, including contributing to the structural integrity of connective tissues and maintaining the moisture, shape, plasticity, and firmness of the vaginal mucosa [12]. ULMW-HA acid has been specifically studied for its ability to cross the intestinal epithelium, with absorption rates directly proportional to its concentration. However, direct evidence for enhanced absorption in humans compared to hyaluronan remains limited [12].

While no prior studies have directly examined these effects in vulvovaginal tissue, this study provides initial clinical support for their relevance. The sustained improvement observed in Group 2 indicates that oral supplementation with BCPs^®^ and other functional ingredients may have a long-lasting physiological impact on the connective tissue, distinguishing it from the symptomatic relief provided by topical products such as moisturizers and lubricants. It is worth noting that the supplement investigated in this study is commercially available and currently patent-pending. It is also notable that oral supplementation was well tolerated, with no reported adverse effects. During the month following discontinuation of the supplement, no symptom rebound, or worsening was observed. Indeed, the supplement is composed of clinically studied ingredients, including specific BCPs^®^ (Verisol^®^), ULMW-HA, a botanical powder mix (*Astragalus membranaceus* and *Centella asiatica*) and a nucleotide blend (5′-AMP, 5′-CMP, 5′-UMP, 5′-GMP, 5′-IMP), and it is produced under GMP- and FSSC 22000-certified conditions, in addition to being free from allergens and artificial colorants. These are important considerations given that many GSM treatments—particularly topical products—may be poorly tolerated or associated with low adherence [21].

Despite the encouraging results, several limitations must be acknowledged. First, the small sample size (n = 20) reduces the statistical power and generalizability of the findings. Second, the lack of a placebo or oral-supplementation-only group prevents definitive conclusions about the individual effects of each treatment component. Furthermore, the absence of histological or objective imaging endpoints limits insight into underlying tissue-level changes. Third, the shorter follow-up suggests that patients should ideally be re-assessed at 6 months or even 12 months after the completion of the last RF session. Therefore, future studies should use larger sample sizes and include three-arm designs (RF only, supplement only, and combination) to dissect the contributions of each intervention. Longer follow-up periods and the inclusion of tissue biomarkers or histological analysis would be valuable in confirming and extending these findings.

Taken together, this study provides preliminary evidence that oral supplementation may enhance and sustain the effects of RF therapy in GSM. While RF alone was associated with short-term benefits, its effect appears to wane over time. The addition of systemic supplementation may offer a promising integrative approach, and further research is warranted to validate these findings in larger, well-controlled studies.

## 5. Conclusions

The combination of vulvovaginal RF therapy with oral supplementation containing BCPs^®^, ULMW-HA, and other functional ingredients may enhance and prolong symptom relief in postmenopausal women with GSM compared to RF therapy alone. Although preliminary, these findings support the feasibility of an integrated treatment approach and warrant further investigation in larger, placebo-controlled trials to confirm efficacy, safety, and durability of response.

## 6. Patents

The present investigation allows the registration of the patent-pending invention “Pinkcare^®^” (ITALY-PATENT OF INVENTION Question N. 102024000014935 of 28 June 2024—Pharmaceutical or nutraceutical composition for prevention and/or treatment of uro-gynecological disorders—Erbozeta S.p.a.). Chiara Pastorelli and Roberto Zavaglia are co-inventors of the patent-pending invention “Pinkcare^®^”.

## Figures and Tables

**Table 1:** Descriptive characteristics of the study population.

Characteristic	Group 1 n = 10 (50%) ^1^	Group 2 n = 10 (50%) ^1^	*p*-Value ^2^
Age (years)	56 (53, 58)	56 (52, 58)	0.7
BMI (kg/m^2^)	25.1 (24.6, 25.7)	25.6 (23.5, 27.8)	0.6
Educational level			0.7
Middle-school diploma	2 (20.0%)	4 (40.0%)	
High-school diploma	5 (50.0%)	4 (40.0%)	
University degree	3 (30.0%)	2 (20.0%)	
Number of pregnancies	2 (1, 3)	2 (1, 3)	0.5
Age at menopause (years)	51 (50, 51)	50 (50, 51)	0.8
Marital status			0.9
Unmarried	1 (10.0%)	2 (20.0%)	
Married	7 (70.0%)	7 (70.0%)	
Separated/divorced	2 (20.0%)	1 (10.0%)	

Group 1: RF alone. Group 2: RF and oral supplementation. Abbreviations: BMI, body mass index. ^1^ Median (IQR); n (%). ^2^ Wilcoxon rank sum test; Fisher’s exact test.

## Data Availability

All data generated or analyzed during this study are included in this article. Further enquires can be directed to the corresponding author.

## Supplementary Materials

The following supporting information can be downloaded at: https://www.mdpi.com/article/10.3390/jcm14113656/s1: Table S1: Subjective symptoms and objective signs scores at each specified time point for patients treated with vulvovaginal radiofrequency only (Group 1) and vulvovaginal radiofrequency + oral food supplementation with BCPs^®^ and other functional ingredients (Group 2).

## Abbreviations

The following abbreviations are used in this manuscript:

GSM	Genitourinary syndrome of menopause
RF	Radiofrequency
BCP	Bioactive collagen peptides
ULMW HA	Ultra-low-molecular-weight hyaluronic acid
BMI	Body mass index
IQR	Interquartile range
