# Supplementary material for: Oral Collagen Peptides and Vulvovaginal Radiofrequency Therapy for Genitourinary Syndrome of Menopause: A Pilot Randomized Study
Source: J Clin Med. 2025 May 23;14(11):3656. doi: 10.3390/jcm14113656 (PMC12156920; doi:10.3390/jcm14113656)
Supplement: Supplementary file 1 [file jcm-14-03656-s001.zip › jcm-3592965-supplementary.pdf]

Table S1. Subjective symptoms and objective signs scores at each specified time points in patients treated with vulvo-vaginal radiofrequency only (Group 1) and vulvo-vaginal radiofrequency + oral food supplementation with BCP® and other functional ingredients (Group 2).

	Group 1 Vulvo-vaginal Radiofrequency			Group 2 Vulvo-vaginal Radiofrequency + food supplement		
	T0	T1	T2	T0	T1	T2
Subjective symptoms						
**Vaginal dryness**	3.00 (2.00 – 3.00)	2.00 (2.00 – 2.00)	2.00 (1.00 - 2.00)	3.00 (2.25 – 3.00)	1.00 (1.00 – 2.00)	1.00 (0.25 – 1.00)
**Dyspareunia**	3.00 (2.00 – 3.00)	1.00 (1.00 – 1.75)	2.00 (1.25 – 2.00)	3.00 (2.00 – 3.00)	1.00 (0.25 – 1.00)	0 (0 – 1.00)
**Irritation/Burning/Itching**	2.00 (2.00 – 2.00)	1.00 (1.00 – 1.00)	1.00 (1.00 – 1.00)	2.00 (2.00 – 2.75)	0 (0 – 1.00)	0 (0 – 0)
**Dysuria**	1.50 (1.00 – 2.00)	0 (0 – 1.00)	1.00 (0 – 1.00)	2.00 (2.00 – 3.00)	0 (0 – 1.00)	0 (0 – 0)
**Bleeding with sexual activity**	1.00 (1.00 – 1.00)	0 (0 – 0)	0 (0 – 0)	1.00 (1.00 – 2.00)	0 (0 – 0)	0 (0 – 0)
**Total**	10.50 (8.00 – 11.00)	4.00 (3.25 - 5.75)	5.50 (3.25 – 6.00)	11.00 (10.25 – 13.75)	3.00 (1.50 – 4.00)	1.00 (1.00 – 2.00)
Objective signs						
**Elasticity**	2.00 (2.00 – 3.00)	1.50 (1.00 – 2.00)	2.00 (1.25 – 2.00)	3.00 (2.00 – 3.00)	1.00 (0 – 2.00)	1.00 (1.00 – 1.75)
**Vaginal folds**	2.00 (2.00 – 3.00)	2.00 (1.00 – 2.00)	2.00 (1.00 – 2.00)	2.50 (2.00 – 3.00)	1.00 (1.00 – 2.00)	1.00 (1.00 – 1.00)
**Fluid secretion**	2.00 (2.00 – 3.00)	1.00 (1.00 – 1.00)	1.00 (1.00 – 1.75)	2.50 (2.00 – 3.00)	1.00 (0 – 1.00)	0 (0 – 0)
**Epithelial thickness**	2.00 (1.25 – 3.00)	1.50 (1.00 – 2.00)	2.00 (2.00 – 2.00)	2.50 (2.00 – 3.00)	1.00 (1.00 – 1.75)	1.0 (1.0 – 1.0)
**Moisture**	2.00 (2.00 – 3.00)	1.00 (1.00 – 1.00)	1.00 (1.00 – 1.00)	2.50 (2.00 – 3.00)	1.00 (0 – 1.00)	0 (0 – 0)
**Color of tissue**	2.00 (2.00 – 3.00)	1.50 (1.00 – 2.00)	2.00 (1.00 – 2.00)	2.50 (2.00 – 3.00)	1.00 (0 – 1.75)	1.00 (0.25 – 1.00)
**Total**	12.00 (11.25 – 18.00)	8.50 (6.25 – 10.00)	10.00 (7.25 – 10.75)	15.50 (12.00 – 18.00)	5.50 (2.25 – 8.75)	4.00 (2.50 – 4.75)
